# Eikenella Corrodens Vertebral Osteomyelitis in a Young Patient With Type I Diabetes Mellitus

**DOI:** 10.7759/cureus.9553

**Published:** 2020-08-04

**Authors:** Kushal Ranabhat, Sabita Bhatta, Raj Kumar Bhatta, Yogesh Acharya

**Affiliations:** 1 Internal Medicine, Hurley Medical Center, Flint, USA; 2 Pediatrics, Woodhull Medical Center, New York City, USA; 3 Neurosurgery, Postgraduate Institute of Medical Education and Research, Chandigarh, IND; 4 Vascular and Endovascular Surgery, Western Vascular Institute, Galway, IRL

**Keywords:** eikenella corrodens, osteomyelitis, spine, vertebra, diabetes mellitus, type i, case report

## Abstract

Vertebral osteomyelitis is an uncommon variant of osteomyelitis. Although *Staphylococcus* and/or *Streptococcus* are commonly associated, alternate pathogens have been implicated in vertebral osteomyelitis, especially in endemic areas and/or immunocompromised patients. Here, we present a case of a young African American female with type I diabetes mellitus who presented to us with worsening back pain. The MRI lumbar spine was suggestive of vertebral osteomyelitis involving the right facet joint of the fifth lumbar (L5) and the first spinal (S1) joint and a significant narrowing of the thecal sac at the L4-L5 vertebral level with an anterior epidural abscess. The patient was started on empirical antibiotics, and surgical intervention was performed with L4-L5 laminectomy and extraction of the epidural abscess. Her pus culture showed *Eikenella corrodens *as a possible cause of vertebral osteomyelitis. She had an uneventful recovery after two weeks of antibiotics (intravenous ceftriaxone) therapy.

## Introduction

Vertebral osteomyelitis or spinal osteomyelitis is an uncommon variant of osteomyelitis, causing 3% to 5% of all osteomyelitis each year [1]. Direct inoculation is not common, but hematogenous spread from other infectious foci can directly spread to the vertebra. Vertebral osteomyelitis could lead to severe complications, like motor weakness, paralysis, or meningitis, with a high mortality rate (11% per year) [1,2]. 

One of the alternate organisms implicated in vertebral osteomyelitis is *Eikenella corrodens*, a fastidious facultative anaerobic gram-negative rod, and a normal flora of the oral cavity, nasopharynx, gastrointestinal, and urogenital tract [3]. They are an unusual cause of vertebral osteomyelitis and are usually missed in the routine aerobic cultures due to the anaerobic properties and slow-growing nature [4]. *E. corrodens* were initially associated with ‘fight-bite’ injuries; however, they are capable of causing various superficial skin infections to more serious systemic infections with or without risk factors [5-7]. Here, we present a case of a young African American female with worsening back pain, who was found to have *E. corrodens* vertebral osteomyelitis.

## Case presentation

A 25-year-old African American female with a past medical history of type I diabetes mellitus presented to our emergency room (ER) with four weeks of progressive back and bilateral (b/l) leg pain with numbness. Her symptoms worsened in the past two days with constant sharp back pain, 10/10 in intensity, severely limiting her movement. The pain migrated to both her legs, and it was associated with numbness and tingling sensation. She also complained of subjective fever and chills for the past one week. She did not have nausea, vomiting, diarrhea, decreased appetite, weight loss, chest pain, cough, leg weakness, bowel and bladder incontinence, and urinary retention. There was no history of trauma, substance abuse, and recent dental procedures. 

She visited our ER three weeks ago for back pain, when she was treated for musculoskeletal back pain with ketorolac (15 mg IV once) and hydrocodone/acetaminophen (5/325 mg oral once). She had a resolution of the symptoms within an hour and was discharged home with hydrocodone/acetaminophen (5/325 mg oral) as needed daily for seven days. She reported taking her pain medication every day without any improvement.

On examination, her weight was 98.8 kg (body mass index 35.2 kg/m^2^). There was no abnormality in her respiratory, cardiac, abdominal, and neurovascular examination. The patient could not walk due to the pain. On musculoskeletal examination, her range of motion of hip was limited due to the associated pain. There was spinal point tenderness at the fifth lumbar (L5) and the first spinal (S1) area with straight leg raising test positive in b/l lower limbs. 

She was investigated with the differential diagnosis of vertebral osteomyelitis, discitis, prolapsed intervertebral disc, musculoskeletal sprain, degenerative disease of vertebrae, and spinal cord tumor. 

Her labs showed mild leukocytosis (13,500 cells per cu. mm, ref: 4,000 and 11,000 cells per cu. mm) with 88% neutrophilia (ref: 50%-70%). The erythrocyte sedimentation rate (ESR) was 106 mm/hr (normal 0-20 mm/hr) and C-reactive protein (CRP) 15.96 mg/L (normal 0.00-10.00 mg/L). 

She was managed with dexamethasone (10 mg oral once), hydrocodone/acetaminophen (5/325 mg oral six hourly), and ketorolac (30 mg intramuscular once). She was admitted to the clinical decision unit overnight to be evaluated by the neurology unit. 

**Figure 1:**
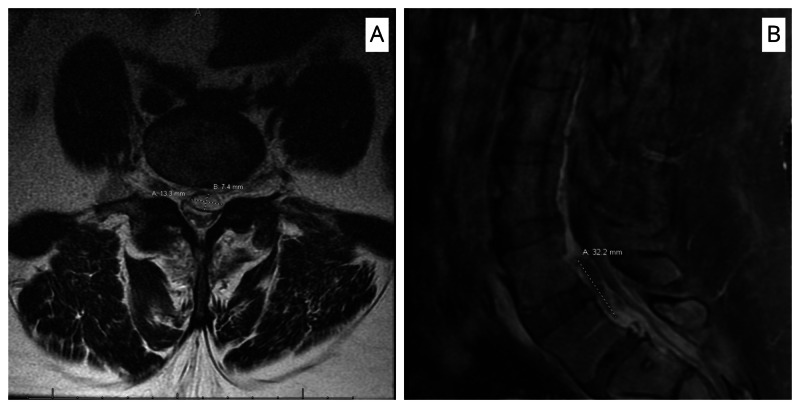
MRI scan (T1 with contrast) of the spine showing epidural abscess measuring 32.2 × 13.3 × 7.4 mm posterior to L4-L5 vertebral body with compression of thecal sac. (A) Axial section. (B) Sagittal section.

Her MRI lumbar spine showed vertebral osteomyelitis involving the right facet joint of L5-S1 with moderate epidural enhancing granulation tissue and anterior epidural abscess measuring 32.2 × 13.3 × 7.4 mm (Figure 1). There was a significant narrowing of the thecal sac at the L4-L5 vertebral level.

The patient was started on empirical antibiotics with vancomycin (15 mg/kg daily) and piperacillin-tazobactam (4,500 mg IV six hourly). She was admitted to neurosurgical floors for neurological assessment every two hours. Surgical intervention with L4-L5 laminectomy and extraction of the epidural abscess was performed the next day. Anaerobic and aerobic cultures were sent, which grew *E. corrodens* after four days.

The infectious disease team descaled antibiotics to ceftriaxone (2 gm IV every 12 hours) after the culture sensitivity result. The patient was admitted to an inpatient rehabilitation center for regular physical and occupational therapy. Her inflammatory markers (ESR and CRP) normalized, and her clinical symptoms showed gradual improvement. She was discharged home after two weeks of inpatient rehabilitation with ceftriaxone. 

At the regular follow-up after a month, she had complete resolution of symptoms and no further complaints. 

## Discussion

Vertebral osteomyelitis is a dreaded infection of the spine. The global incidence of vertebral osteomyelitis is estimated to be between one and seven per 100,000 populations, and the incidence in the US is around four to five cases per 100,000 populations [1]. Hematogenous spread is the conventional route, but direct traumatic inoculation is possible [2]. *Staphylococcus* and/or *Streptococcus* are usually implicated as the causative agents, but alternate pathogens have been isolated from endemic areas and/or immunocompromised patients. The common risk factors include old age, immunosuppression status, diabetes, prolonged steroid use, cancers, malnutrition, and intravenous drug abusers [8]. *E. corrodens* is an uncommon cause of vertebral osteomyelitis, and we could trace only a few cases of distinctive *E. corrodens *vertebral osteomyelitis in the literature [5,9-15]. 

Back pain is the commonest presentation in vertebral osteomyelitis, which localizes as the disease progresses (58% lumbar spine, 30% thoracic spine, and 11% in the cervical spine). Fever will be present in only around 35%-60% of cases [16]. Spine tenderness is possible but has a low sensitivity (20%) [17]. Approximately one-third of the patients will have associated neurological impairment or radiculopathy, ranging from sensory loss, numbness, tingling, sensation, or weakness [16]. Our patient had progressive back pain, b/l leg pain, and numbness, without neurological deficit. 

Vertebral osteomyelitis has a broad, nonspecific presentation that is likely to delay the diagnosis [8]. Complete blood count (CBC) has low sensitivity, whereas the inflammatory markers, like ESR and CRP, have high sensitivity (94%-100%) [2]. Our patient had mild leukocytosis with neutrophilia with elevated ESR and CRP. However, they are nonspecific and only serve as initial workups and/or markers for treatment success. Blood culture is considered routinely in patients with fever and back pain, but *E corrodens *cannot be isolated in the routine aerobic cultures, requiring a high index of suspicion for anaerobic culture. A routine aerobic and anaerobic culture is necessary to avoid missing any alternate pathogens. 

Radiological investigation plays an essential role in the diagnosis of vertebral osteomyelitis, and their utility also lies in ruling out alternate pathologies [16]. MRI is preferred for suspected spinal infection due to the high accuracy [2,18]. CT is better than plain radiographs, but they can miss the soft tissue and bony extent in the early and advanced cases [18]. Percutaneous aspiration or biopsy can be useful to isolate the organism to direct the antibiotic therapy or confirm the diagnosis when blood cultures are negative despite imaging suggestive of vertebral osteomyelitis [2]. We performed an MRI lumbar spine, which was indicative of vertebral osteomyelitis with an epidural abscess. 

Antibiotic therapy is the cornerstone of the management strategy, and there are different regimens. *E. corrodens *are susceptible to broad-spectrum cephalosporins and fluoroquinolones, like ceftriaxone and levofloxacin [19]. We treated our patient with ceftriaxone for six weeks (after initial empirical treatment with vancomycin and piperacillin-tazobactam) once the culture sensitivity result was out. Most of the patients with *E. corrodens* have been treated with cephalosporins (second or third generations) for six weeks. In some cases, the overall treatment has been extended to eight weeks [12]. 

Surgical management is reserved for neurological impairment, significant vertebral destruction leading to spine instability, large epidural abscess, and failure of medical management with nonresolving intractable back pain [2].

The overall prognosis of vertebral osteomyelitis has improved after the discovery of antibiotics. However, some patients may require multiple procedures. Notably, surgery can have various complications, ranging from mild to moderate neurological deficits and functional impairment to severe complications, like permanent neurological deficits and paralysis, leading to poor quality of life. Recovery could be prolonged, and rehabilitation with continuous follow-up with serial scans may be needed.

*E. corrodens* has been previously isolated from osteomyelitis in the feet of diabetic patients [20]. We did not find an apparent focus of infection in our patient, but given her diabetic status, our patient was at obvious risk. Therefore, we must have a high index of suspicion for vertebral osteomyelitis in young patients with associated risk factors, like diabetes, who present with progressive back pain and/or neurological deficit. Early diagnosis, appropriate management with antibiotics, and rehabilitation enhance the recovery and reduce complications.

## Conclusions

*E. corrodens *can cause vertebral osteomyelitis in young patients, especially in the presence of risk factors like diabetes mellitus. There is a likely chance that the *E. corrodens* can be missed in the usual aerobic culture due to its slow growth in the anaerobic medium. Therefore, it is important for physicians to perform an aerobic as well as anaerobic culture to avoid missing any alternative pathogens associated with vertebral osteomyelitis. A high degree of vigilance and strong clinical suspicion within the background of associated risk factors can be crucial for early diagnosis of *E. corrodens* vertebral osteomyelitis. Rapid diagnosis and commencement of appropriate antibiotics prevent subsequent complications and improve recovery.
